# Bioscaffold-Induced Brain Tissue Regeneration

**DOI:** 10.3389/fnins.2019.01156

**Published:** 2019-11-07

**Authors:** Michel Modo

**Affiliations:** ^1^McGowan Institute for Regenerative Medicine, University of Pittsburgh, Pittsburgh, PA, United States; ^2^Center for the Neural Basis of Cognition, University of Pittsburgh, Pittsburgh, PA, United States; ^3^Department of Bioengineering, University of Pittsburgh, Pittsburgh, PA, United States; ^4^Department of Radiology, University of Pittsburgh, Pittsburgh, PA, United States

**Keywords:** extracellular matrix, stroke, regeneration, tissue repair, biodegradation, biomaterial, scaffold, physical therapy

## Abstract

Brain tissue lost after a stroke is not regenerated, although a repair response associated with neurogenesis does occur. A failure to regenerate functional brain tissue is not caused by the lack of available neural cells, but rather the absence of structural support to permit a repopulation of the lesion cavity. Inductive bioscaffolds can provide this support and promote the invasion of host cells into the tissue void. The putative mechanisms of bioscaffold degradation and its pivotal role to permit invasion of neural cells are reviewed and discussed in comparison to peripheral wound healing. Key differences between regenerating and non-regenerating tissues are contrasted in an evolutionary context, with a special focus on the neurogenic response as a *conditio sine qua non* for brain regeneration. The pivotal role of the immune system in biodegradation and the formation of a neovasculature are contextualized with regeneration of peripheral soft tissues. The application of rehabilitation to integrate newly forming brain tissue is suggested as necessary to develop functional tissue that can alleviate behavioral impairments. Pertinent aspects of brain tissue development are considered to provide guidance to produce a metabolically and functionally integrated *de novo* tissue. Although little is currently known about mechanisms involved in brain tissue regeneration, this review outlines the various components and their interplay to provide a framework for ongoing and future studies. It is envisaged that a better understanding of the mechanisms involved in brain tissue regeneration will improve the design of biomaterials and the methods used for implantation, as well as rehabilitation strategies that support the restoration of behavioral functions.

## Introduction

Acute brain injury and progressive neurodegenerative disease represent some of the most challenging medical conditions. The financial burden of these conditions in the US alone amounts to nearly $800 billion per year (Gooch et al., 2017). With an aging population, this cost will continue to rise, especially considering the lack of effective treatment options. There are currently no approved therapies to limit or revert cell loss. In a few conditions, such as Parkinson’s disease, pharmacological agents can compensate for the loss of a specific neurotransmitter (e.g., dopamine). However, the continued cell loss due to neurodegeneration is not reversed. This continued loss of cells produces a tissue atrophy that gradually shrinks brain tissue. In contrast to tissue atrophy, acute brain injuries, such as a stroke and penetrating traumatic brain injuries, produce a volumetric tissue loss that is characterized by cavitation, i.e., cell and matrix loss (Moreau et al., 2012). Tissues surrounding this cavitation are also damaged and typically undergo an acute and sub-acute neuronal loss associated with reactive gliosis and angiogenesis. Atrophy in peri-cavity damaged tissue can also occur. Pharmacological therapies, such as neuroprotective agents, are primarily focused on rescuing acutely dying neurons, whereas anti-inflammatory agents target the immune system’s response to the inflicted damage, aiming to reduce secondary tissue damage (Neuhaus et al., 2017). Neither approach replaces lost cells or tissues.

In contrast to these pharmacological interventions, biological interventions aim to replace cellular components that have been lost (Modo et al., 2018). For example, in the case of Parkinson’s disease, ectopic transplantation of dopaminergic cells attempts to restore the local neurotransmitter tone, whereas in Huntington’s disease the goal is to replace lost neurons and to integrate these new neurons into existing neuronal circuitry (Barker and TRANSEURO consortium, 2019). In the case of acute brain injuries, two paradigms have found clinical translation, with one focused on influencing the immune response using intravenous or intra-arterial injections of mesenchymal stem cells during the acute phase of injury, whereas the other focuses on intracerebral implantation of cells into damaged tissue in the sub-acute to chronic phase by supplementing endogenous repair mechanism (Ghuman et al., 2018; Borlongan, 2019). Emerging evidence suggests that brain regeneration is feasible if the appropriate conditions are engineered (Ghuman et al., 2018; Nih et al., 2018; Modo and Badylak, 2019). An endogenous repair response to tissue damage and injury is crucial for this process and replicates certain aspects of peripheral wound healing. Herein, we review putative mechanisms involved in brain tissue regeneration and contrast these mechanisms with peripheral soft tissue regeneration following injury.

## Tissue Repair Versus Regeneration

Progressive neurodegeneration or acute brain injury lead to cell death by several mechanisms, including apoptosis, necrosis, oncosis, autophagy, and pyroptosis. The resulting cell debris activates resident immune cells, such as microglia, and is associated with reactive astrocytes that change morphology and function (Donnelly and Popovich, 2008; Chapman et al., 2015; Gabel et al., 2016). These cell and tissue responses mitigate the impact on surrounding healthy cells and tissues. In the case of penetrative and ischemic injuries, liquefactive necrosis occurs in the core of the injured tissue, with an associated loss of the tissue structure itself. The liquefactive debris, including cell debris and the disrupted extracellular matrix, are removed, leaving behind a tissue cavity. These events occur in up to 94% of patients with an ischemic stroke (Moreau et al., 2012). Still, tissue cavitation remains a poorly understood phenomenon with location and time post-injury being key factors. Some species, such as rat and human, are more prone to this process than others, such as mice (Surey et al., 2014). Inflammation may play a key role in this process and explain differences between species, as well as location (Chung et al., 2018). The liquefactive debris is itself neurotoxic upon permeation into peri-cavity tissue (Zbesko et al., 2018) and might provoke a pro-inflammatory response. The tissue inflammatory response eventually results in a structural barrier in the form of gliosis and scarring that prevents the spread of neurotoxic debris to limit further tissue damage.

In addition to the formation of a glial scar, local astrocytes and oligodendrocyte progenitor cells proliferate to produce an expanded cell population that can support tissue function. A small proportion of these cells may have the potential to replace lost neurons (Donnelly and Popovich, 2008; Chapman et al., 2015; Gabel et al., 2016), but evidence for this remains controversial. The local vasculature also responds to tissue damage with the formation of new small blood vessels that improve nutrient supply to hypoxic tissue containing dying or at-risk cells (Manoonkitiwongsa et al., 2001; Senior, 2001).

**FIGURE 1:**
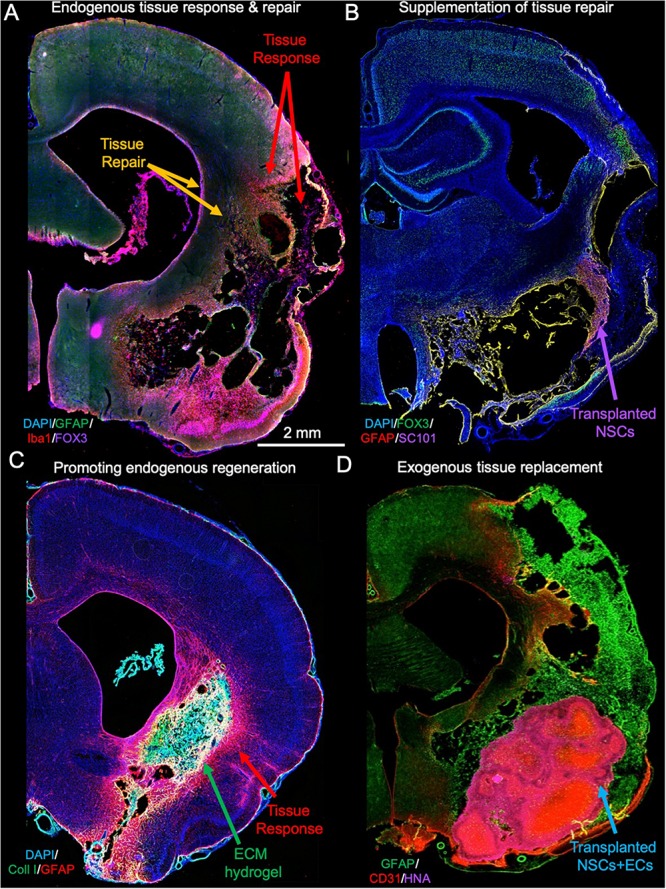
Tissue response, repair, and regenerative response. **(A)** After acute brain injuries, such as stroke, astrocytes and microglia get activated to respond to tissue damage that at its core eventually produce a loss of cells, as well as the extracellular matrix, leaving behind a cavity. The glial scar is aimed at providing structural support but also preventing neurotoxic fluid to permeate into the peri-infarct tissue. In addition to this tissue response, the brain mounts reparative activities, which include an upregulation of neurogenesis in the subventricular zone. **(B)** To supplement the endogenous repair response, neural stem cells (NSCs) can be transplanted into damaged tissue where these differentiate and integrate with host cells, but they also increase local angiogenesis providing a better blood supply to poorly perfused tissues (Smith et al., 2012). **(C)** In contrast to cell transplantation, implantation of inductive extracellular matrix (ECM) bioscaffolds promotes a restoration of tissue inside the lesion cavity from endogenous cells that invade the material (Ghuman et al., 2018). **(D)** An alternative approach to tissue regeneration using an inductive bioscaffolds is to implant a mixture of NSCs and ECs that spontaneously organize into a vascular and neuropil compartment, potentially accelerating the restoration process and overcoming the potential limitation of the reservoir of endogenous cells (Nicholls et al., 2015).

The brain possesses a neurogenic potential that is involved in maintaining tissue homeostasis during normal aging. However, recent evidence suggests that neurogenesis in adult human brain is very limited (Kempermann et al., 2018). After injury or during ongoing neurodegeneration, neurogenic regions, such as the subventricular zone (SVZ) upregulate the proliferation of progenitor cells, which then migrate toward the area of damage (Figure 1A) (Lindvall and Kokaia, 2015). This repair response is still active more than 1 year after a stroke (Kazanis et al., 2013), but survival and integration of newly generated cells is generally considered poor (Magavi et al., 2000; Arvidsson et al., 2002), potentially due to a non-permissive environment. New blood vessels might serve as a guidance conduit toward areas of damage (Kojima et al., 2010). Intraparenchymal cell transplants aim to supplement this endogenous tissue repair response (Figure 1B), without replacing lost tissue (Smith et al., 2012). For the present review, we define tissue repair to consist of the changes that occur in and around damaged tissue without the replacement of any lost brain tissue (Table 1).

**TABLE 1:** Terminology used to define different aspects associated with tissue regeneration.

**Terminology**	**Description**
Angiogenesis	The process of a new artery branching out from an existing artery
Anastomosis	Establishment of a cross-connection between two blood vessels to create a new supply network
Arteriogenesis	The process of creating a new artery, including engineering of engraftable arteries
Axonogenesis	The formation of new axons
Behavioral Recovery	Improvements observed in solving behavioral tasks (e.g., walking)
Cell Replacement	Replacement of cells lost due to injury either through endogenous processes or through cell implantation
*De novo* tissue	Tissue that has newly formed where there was none, includes tissue that is established through endogenous means (i.e., regeneration) or through implantation (e.g., cells plus bioscaffold)
Developing tissue	Tissue that is undergoing development, i.e., cells invade and mature
Functional Recovery	Re-establishment of an electrophysiological connectivity (e.g., evoked potential between motor and somatosensory cortex)
Functional Tissue	The establishment of a tissue that supports brain activity (e.g., metabolic response to glucose)
*In situ* tissue engineering	Creating a new tissue through use of cells and biomaterials in the location where it is needed
Neovascularization	Formation of new blood vessels in a tissue void of vasculature, as in the case of tissue regeneration
Neuro/Gliogenesis	The formation of new neurons and glial cells
Tissue Response	Biological activity in tissue that responds to injury (e.g., glial scarring, cavitation)
Primitive tissue	Tissue that is not completely formed (e.g., tissue containing cells, but lacking appropriate cytoarchitecture or connectivity)
Revascularization	Re-establishing a vascular supply in a tissue that was lacking it, as in poorly perfused peri-infarct tissue
Synaptogenesis	The formation of new synapses
Tissue construct	A pre-formed *ex vivo* tissue that can be implanted
Tissue neogenesis	Newly developing tissue in a cavity, as opposed to damaged tissue being repaired to establish a functional tissue again
Tissue Regeneration	Establishment of a new tissue in a cavity through endogenous cells, including axonal connections
Tissue Repair	Cellular response to tissue damage trying to restore function (e.g., neurogenesis, angiogenesis)
Tissue Restoration	Establishment of a new tissue in a cavity through endogenous or exogenous means
Tissue engineering	Use of cells and biomaterials to create a new tissue *in vivo*, *ex vivo*, or *in vitro*
Vasculogenesis	The process of creating a new vasculature, including veins
Veterate tissue	Organ tissue that is established and remained after injury (e.g., peri-infarct tissue), as opposed to partially formed or newly forming tissue

Tissue regeneration or replacement of lost tissue requires the infiltration and organization of functional neurons, glial cells and support structures (e.g., vascularization). Infiltration of cells into the lesion cavity requires the presence of a matrix substrate to facilitate cell migration. Provision of inductive bioscaffolds, such as those composed of extracellular matrix (ECM) derived from decellularized tissues, can facilitate endogenous tissue regeneration in the brain (Figure 1C). Other materials, such as cross-linked hyaluronic acid might serve the same function (Nih et al., 2018). *In vitro* morphogenesis of brain tissue, i.e., organoids, have also been reported to spontaneously arrange into structures that resemble cortical layers. This further indicates that there is an inherent potential in brain cells to structurally organize into tissue if the appropriate conditions are provided (Mansour et al., 2018). A further example of this tissue morphogenesis is provided by the co-implantation of a dense mixture of NSCs and endothelial cells (ECs) into a stroke cavity (Nicholls et al., 2015). Implantation of essential brain tissue components (i.e., NSCs, ECs, ECM) could therefore provide an alternative or supplementation to endogenous tissue regeneration (Figure 1D).

## The Brain’s Failure to Regenerate

Ramon y Cajal professed that “*In adult centers the nerve paths are something fixed, ended, immutable. Everything may die, nothing may be regenerated. It is key for the science of the future to change, if possible, this decree*” (Ramon y Cajal, 1928). The discovery of neurogenic zones in the adult mammalian brain and their participation in response to tissue injury partially refuted this long-held dogma that neurons in the brain cannot be replaced (Kempermann et al., 2018). However, the belief that the brain cannot regenerate (i.e., form new functional tissue) has mostly remained unchallenged (Fry, 2001; Bechmann, 2005; Illis, 2012). By many, regeneration of brain tissue is considered the ultimate challenge for regenerative medicine (Obernier et al., 2014): by others, it is considered a biological impossibility (Price, 2011). Essentially, two main arguments are postulated as to why brain tissue regeneration is impossible: (1) The central nervous system of adult mammals is an inhibitory environment that “irreversibly” seals off tissue cavities to protect the remaining brain and consequently prevent tissue regeneration; and (2) The brain arises through a complex interplay between cells during development to form long-distance connections within and between brain regions, which cannot be recreated in the adult. Both viewpoints have merit, but do not consider the possibility that certain engineering strategies could overcome the scarring around the tissue cavity, or that functional brain tissue can develop via alternative mechanisms from normal brain development. Indeed, most tissue regeneration (e.g., liver) utilizes certain developmental processes, but does not strictly recapitulate fetal development.

**FIGURE 2:**
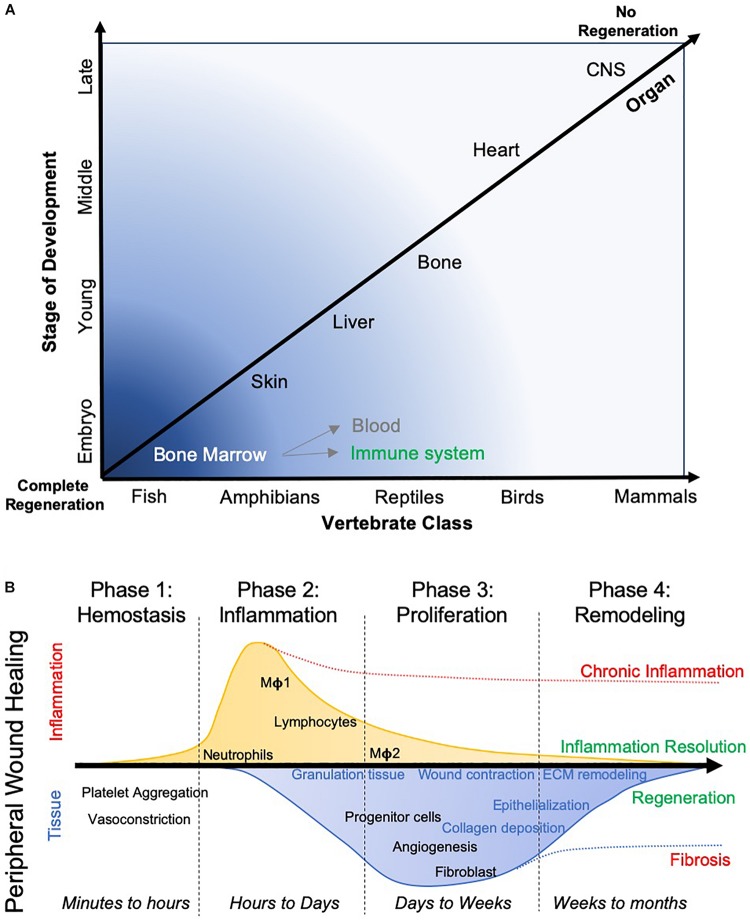
Evolutionary aspects of tissue regeneration. **(A)** The capacity of different species to regenerate different organ tissue over their lifespan widely varies. Anatomical complexity of the species, as well as tissues, is a major factor in their ability to regenerate. Lower species more readily regenerate more complex tissue, whereas more complex species, such as mammals, are only able to regenerate very few organs. It is pertinent to contrast a replenishing of blood and immune system from the bone marrow, which essentially restitutes single cells, from constructing a tissue that involves multiple cell lineages and the deposition of ECM to create a geometric arrangement of cells. **(B)** In wound healing, where a cut in a tissue, such as the skin, restores tissue integrity, typically follows 4 phases. These phases are characterized by a major difference in inflammation, which peaks in phase 2, but also by changes in tissue characteristics, with the deposition of a transient granulation tissue that is being remodeled by the infiltration of host tissue cells. The formation of blood vessels and epithelization of the tissue are further key events required to ensure that a seamless wound closure occurs. Depending on which process is interrupted, a wound breakdown can occur or scarring occurs that limits tissue function. This process can take months to complete.

When considering tissue regeneration across multiple organ systems and species, it is evident that tissue regeneration is reduced as a function of age and the complexity of the organ system (Figure 2A). Less complicated organ systems, such as the hematopoietic system, readily replenish. With increasing tissue complexity, as evidenced in most solid organs, there is less replenishing of cells during homeostasis, less cell replacement after injury and more limited regeneration after tissue loss compared to the hematopoietic system. A notable exception is the liver, which can completely regenerate from only 25% of remaining tissue (Michalopoulos and DeFrances, 1997). However, the reasons for a disparate regenerative potential of tissues and organs remains unknown at the present time.

A differential capacity to regenerate complex tissue exists across the evolutionary ladder (Tanaka and Ferretti, 2009; Ferretti, 2011), with species such as zebrafish and newts able to “regenerate” brain tissue after ablation (Kroehne et al., 2011; Urata et al., 2018). Inflammation at the site of injury and the invasion of macrophages are increasingly emerging as a key signaling axis to initiate and regulate this tissue regeneration (Kyritsis et al., 2012; Godwin et al., 2013). In the brain, the presence of neurogenic regions is considered a *conditio sine qua non-for* tissue repair and regeneration (Ferretti, 2011). Indeed, the size of the neurogenic reservoir directly correlated with the degree of tissue regeneration in carp (Kirsche and Kirsche, 1961). Large neurogenic regions persist into zebrafish adulthood, which respond to ablations in nearby brain regions (Kizil et al., 2012). The requirement for adequate neurogenic tissue would suggest that the proximity to a neurogenic region will be a determining factor in promoting tissue repair and regeneration. Relative distances in larger mammalian brains might hence be more difficult to bridge than those in smaller brains. Alternatively, this required cellular reservoir may be supplied through implantation, as is the case in the transplantation of neural stem cells (NSCs). The neurogenic region’s supply of NSCs and neural progenitors that can participate in tissue repair and regeneration is therefore a crucial element in brain tissue regeneration.

Time is a separate factor to consider, with a “perfect” tissue replacement being a slow process (>1 year) and dependent on wound closure through a tubular arrangement of ependymoglia cells (Urata et al., 2018). In axolotls and newts, true tissue regeneration after a volumetric defect can occur (Amamoto et al., 2016; Urata et al., 2018). However, alterations in cytoarchitecture and a failure to re-establish long-distance connections can happen in regenerated tissue in adult axolotls, even though cells were electrophysiologically appropriate for the tissue (Amamoto et al., 2016). It is nevertheless unclear if an altered cytoarchitectural arrangement will not allow the tissue to function as needed. Anomalies in spontaneously regenerated tissue were evident in axolotls (Amamoto et al., 2016) and zebrafish (Kroehne et al., 2011; Kaslin et al., 2017), whereas adult newt exhibit a more complete structural and cellular regeneration (Urata et al., 2018). Studies in neuro-regenerative species can therefore provide unique insights into the key factors that are required to engineer functional brain tissue regeneration in adult mammals.

## Wound Healing Versus Tissue Regeneration

In adult mammals, healing of the skin is often considered a key example of how adult tissue can regenerate. Typically, four phases are recognized in this “regenerative process”: (1) Hemostasis; (2) Inflammation; (3) Proliferation and tissue growth; and (4) Remodeling and maturation (Figure 2B). During injury to the skin, blood vessels are disrupted and platelets are released in the area of damage (Eming et al., 2014). The adherent platelet plug assumes an amorphous shape covering the lesion. Fibrin acts as “glue” that bridges the edges of tissue, essentially restoring the physical support for the next phase of wound repair, the invasion of inflammatory cells. Neutrophils and mast cells are among the first cells invading the clot and initiate a cascade of cytokines and chemokines that promote further cell invasion (e.g., macrophages), eventually leading to the removal of the cellular debris and the clot. The cytokine milieu also facilitates the recruitment of tissue-specific cells to repopulate the area of damage. During this proliferation phase new tissue is gradually assembled, including a restoration of vascular supply and deposition of tissue-specific ECM. Granulation tissue is formed, but is remodeled and replaced during the tissue maturation process. It is important to note the sequence of events, as these are dependent on each other for a successful wound healing process. A delay in blood clotting or a lack of re-epithelization will result in scar formation and prevent wound healing (Martin and Nunan, 2015).

It has been proposed that the repair of CNS injury resembles peripheral tissue wound healing, with the key difference being that in the CNS an insufficient resolution of pro-inflammatory events occurs (Shechter and Schwartz, 2013). The brain’s response to injury therefore is akin to an unresolved wound healing process, in which a lack of a timely re-epithelization leads to scar formation. Scar formation is commonly seen as the main obstacle to the repair and regeneration of CNS tissues (Silver and Miller, 2004). However, it is also notable that peripheral tissue wound healing typically occurs following an acute disruption of tissue due to a cut or a superficial wound. The injuries typically involve a shallow loss of cell layers (i.e., more of a repair than regenerative response) or a very narrow disruption between layers occurs. Typically, these injuries do not involve a major volumetric loss of tissue. This is comparable to traumatic brain injury caused by penetration of a sharp object, such as a knife or an injection tract. We here argue that this type of injury and its associated repair response is quite different from what is required to regenerate a large volumetric tissue loss, as would occur after a stroke or removal of parts of the liver. The wound healing paradigm is therefore useful to understand how a reparative response is mounting to a particular type of tissue injury, but it fails to account for major tissue loss, which might require additional strategies to re-grow lost tissue.

The best known example of tissue regeneration in adult mammals is liver tissue regeneration (Cordero-Espinoza and Huch, 2018). A liver can regenerate within 5–7 days in a mouse and 8–15 days in humans (Michalopoulos, 2007). Tissue regeneration occurs in cases of volumetric tissue loss, as exemplified by a partial hepatectomy, but not in cases of certain types of liver disease, such as liver cirrhosis, further showing a difference between a tissue repair and regenerative response. Regeneration of tissue occurs from undamaged lobes by creating new tissue rather than expanding existing tissue. Akin to the inflammation phase in wound healing, macrophages resident in the liver (i.e., Kupffer cells), as well as those recruited from the blood, initiate an inflammatory cascade that stimulates hepatocyte progenitor proliferation and migration.

Myofibroblasts deposit matrix at the tissue boundary. This is degradable by matrix metalloproteinases (MMPs) and replaced by ECM deposited from stromal cells that integrate into the matrix. Upon integration, cells differentiate into appropriate phenotypes. The creation of a transitional ECM is similar to the formation of granulation tissue in wound healing and might indicate that remodeling of a temporary matrix is a key component of actual tissue creation. In addition to progenitor cell proliferation, de-differentiation and re-differentiation of stromal cells occurs. This cell pool is an important contributor to re-populate the regenerating tissue (Gilgenkrantz and Collin de I’Hortet, 2018). The extent of tissue regeneration might hence depend on the available cellular pool that can supply sufficient cells to regrow the needed tissue.

However, in the brain the pool of cells being able to replace lost tissue is thought to be restricted to neurogenic regions in the subventricular zone (SVZ) and the subgranular zone (SGZ) of the hippocampus (Ming and Song, 2011). This restricted endogenous neurogenic potential of brain tissue might therefore need to be complemented. Implantation of fetal tissue that can develop into site-appropriate brain tissue potentially provides all the required elements to regenerate lost brain tissue and complement the endogenous tissue repair process.

## Fetal CNS Tissue Transplants and Brain Development

Transplantation of fetal brain tissue is providing a substrate that contains all required elements to construct brain tissue, notably neural cells, a vasculature, microglia, as well as ECM. To ensure optimal survival, post-lesion implantation time and the age of the donor are two crucial variables. Implantable tissue should be derived from the neurogenic period of the developing brain (E11-17 in mice/rats, GW8-30 in humans) (van den Ameele et al., 2014). Earlier tissues are likely to still contain embryonic stem cells that could form teratocarcinomas, whereas more developed tissues will contain partially differentiated neurons, which are likely to die due to axotomy that results in apoptosis. The neurogenic phase is followed by the gliogenic phase (>E18), which produces astrocytes and oligodendrocytes, limiting the yield of neurons required to support function (Sauvageot and Stiles, 2002). The neurogenic tissue hence provides appropriate conditions to produce new brain tissue after implantation into a tissue cavity. A 2–3 weeks post-lesion time frame is considered to be a favorable pro-repair environment for fetal tissue transplants into a cavity, whereas integration and survival at later time points is potentially reduced by the maturation of a glial scar limiting access to host tissue (Grabowski et al., 1994).

Preclinical rodent studies using fetal tissue implanted into the stroke cavity showed the formation of new tissue that filled the cavity (Grabowski et al., 1992b, 1994; Hadani et al., 1992). This fetal tissue developed a vasculature that integrated with the host vasculature and afforded normal metabolic functioning of the tissue (Miyoshi et al., 1995a, b). However, grafts were easily identifiable and distinguishable from host tissue. A tissue mass was created in the cavity, rather than developing a homogenous transition of cytoarchitecture between the host and grafted tissues (Sorensen et al., 1996). A distinct scar separates host and graft highlighting limits to integration (Zeng et al., 1999). Still, implanted tissue fragments matured with neuronal differentiation that led to efferent and afferent axonal projections (Grabowski et al., 1992a, 1993; Sorensen et al., 1996). Although tissue grafts improved behavioral deficits (Grabowski et al., 1995), it remains unclear if this efficacy was due to neuronal connections being formed or if it was mainly due to a trophic factor support reducing secondary degeneration (Mattsson et al., 1997). Nevertheless, these studies demonstrated that new brain tissue can develop in a cavity formed after a stroke.

**FIGURE 3:**
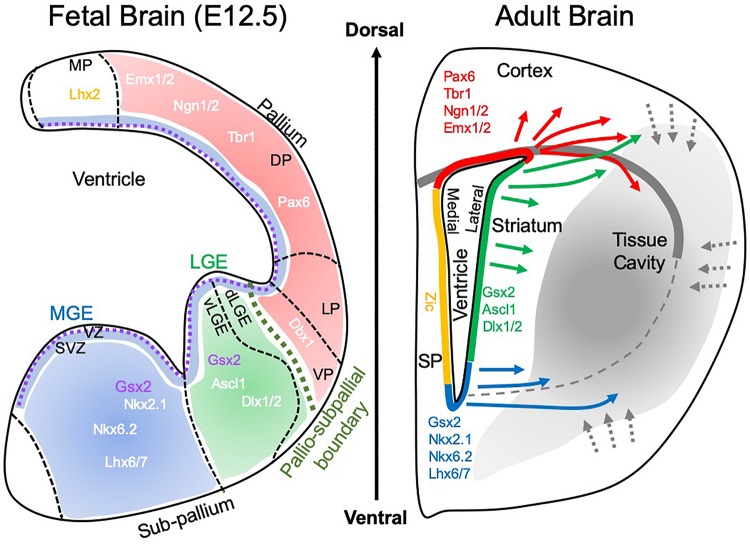
Brain development and tissue repair. During brain development, the pallio-subpallial boundary defines the divide between regions that mature into cortical and striatal tissues. The subventricular zone (SVZ) lies beneath the ventricular zone (VZ) and during development is the birthplace for cells that colonize tissue by migrating along radial glia. Within these tissues, particular gene expressions define the positional specifications of cells to become neurons that characterize the functions of individual regions. The medial ganglionic eminence (MGE) produces striatal interneurons (i.e., calretinin, paravalbumin, calbindin, cholinergic positive), whereas the lateral ganglionic eminence (LGE) is the main source of striatal projection neurons, which constitute 90% of neurons in the striatum. Of these 98% are DARPP-32 positive neurons. A further sub-division of the LGE into the ventral (vLGE) and dorsal (dLGE) has emerged, with vLGE producing a subset of projection neurons. The dLGE is thought to be the main source of interneurons in the olfactory bulb. In contrast, the pallium (a.k.a. telencephalon) is giving rise to the cortex with subdivisions of the ventral pallium (VP), lateral pallium (LP), deep pallium (DP) and medial pallium (MP). These regions produce different subdivision of the cortex, such as the motor cortex and somatosensory cortex. In the adult brain, this positional specification is retained within the subventricular zone (SVZ), the site of adult endogenous neurogenesis. Neurons born along different segments of the SVZ therefore contain a certain positional specification to produce region-specific cells. In response to acute brain injury. These cells respond and migrate through damaged tissue. In the context of tissue regeneration, the SVZ is the main source of cells to replenish lost cells. It remains currently unclear if these cells will cross the pallio-subpallial division that is defined by the lateral corpus callosum in adults. It also remains unknown if cells can change their positional specification and what functional consequence ensue if cells are not expressing a site-appropriate neuron differentiation.

A regional specificity is observed with brain tissue implants. Cortical grafts implanted into the striatum developing as cortical rather than striatal tissue (Wictorin et al., 1991). This result reflects the positional specification of different brain regions during development (Figure 3). Cortical tissues typically develop from the pallium through Pax6-dependent positional specification, whereas the striatum (i.e., caudate-putamen) develop from the lateral and medial ganglionic eminence (LGE and MGE) (Campbell, 2003). The LGE is specified by Gsx2 and Ascl1, whereas the MGE is defined by both Gsx2 and Nkx2.1 (Evans et al., 2012). In the adult, this positional specification is mostly restricted to the SVZ with the dorsal element reflecting the pallial specification (i.e., Pax6 and Emx1/2), the middle section expressing Gsx2 and the ventral part Nkx2.1 (Chaker et al., 2016; Delgado and Lim, 2017). Neurogenesis in the SVZ in the adult therefore produces progenitors that contain some positional specification, which can affect their potential to repair site-appropriate tissue.

## Neural Stem Cells: From Tissue Repair to Regeneration

The differential expression of positional specification in the adult SVZ has profound implications for tissue repair and regeneration. If the neural progenitors are already pre-specified to develop into cortical or striatal phenotypes, their migration across the corpus callosum could direct these into heterologous tissues (Figure 3). Commonly, only the SVZ region affected by damage is mounting a repair response with a transient proliferation of NSCs and a more prolonged activation (>1 year) of neural progenitors (Kazanis et al., 2013). If only a restricted portion of the SVZ is responding to tissue injury, this might limit the type of cells available for tissue regeneration. It remains to be determined if this positional specification can be re-specified upon final differentiation. Although fetal tissue experiments suggest that cortical cells will develop into cortex even if implanted into the striatum (Wictorin et al., 1991), these transplants are considerably different from individual cells migrating and interacting with a striatal environment. It is also unclear what the functional effects of differentiated cortical neurons in striatal tissue would be. Cortical NSCs implanted into the stroke-damaged striatum integrate positionally into peri-infarct tissue, in some cases extensive (17%) neuronal differentiation is observed (Darsalia et al., 2007), whereas in others only very few transplanted cells (<2%) actually become neurons (Smith et al., 2012). It therefore remains unclear to what degree the tissue region influences individual transplanted NSCs. For instance, typically striatal projection neurons are produced by cells from the LGE, whereas striatal GABAergic and cholinergic interneurons are derived from the MGE (Marin et al., 2000). Neuroblasts responding to stroke co-express markers for striatal projection neurons (i.e., Pbx and Meis2), indicating their regional specificity stemming from the LGE region (Arvidsson et al., 2002). Hence, both a contribution of MGE and LGE regions of the SVZ are required to produce a functional striatal tissue. Multiple SVZ regions therefore need to respond to striatal tissue damage to reconstruct functional circuitries. In the case of tissue damage encompassing both striatal and cortical tissue, it is further tantalizing to speculate that cortical and striatal cells would sort themselves into their respective territories to restore a pallio-subpallial boundary. Some local cell proliferation is occurring and participating in the tissue response, for instance, glial scarring, but also by producing a small number of neural progenitors (Buffo et al., 2008; Gabel et al., 2016). Astrocytes from the lesion environment gain the potential to produce neurons *in vitro* (Buffo et al., 2008), but *in vivo* the restrictive non-permissive environment suppresses this potential (Seidenfaden et al., 2006; Costa et al., 2010; Robel et al., 2011). This local response is therefore not considered a major source of neuronal replacement in damaged tissue, but these cells are in a position to rapidly invade a cavity and initiate tissue regeneration.

An enhanced proliferation and mobilization of neuroblasts is likely to be advantageous to promote a more extensive tissue repair, but will be essential to produce a sufficient cell pool to replace lost tissue. Intracerebral infusion of growth factors, such as glial cell line derived neurotrophic factor (GDNF), enhanced striatal neurogenesis in the SVZ, but also improved the survival of cells migrating into the stroke-damaged striatum (Kobayashi et al., 2006). A continued infusion of growth factors, such as EGF or betacellulin (Gomez-Gaviro et al., 2012), into the lateral ventricle can hence increase the proliferation of NSCs and/or neuroblast to improve invasion into the damaged striatum and cortex. However, at present it is unclear if these different SVZ sub-regions can be stimulated separately to control tissue specificity of neural progenitors. Proliferating peri-infarct cells could also provide a source of cells that can be expanded to provide a cellular population that can promote tissue regeneration. Stimulation of the progenitor pool prior to tissue regeneration could be important to ensure that a sufficient invasion of progenitors is available in the damaged tissue to afford invasion into the cavity to replace lost tissue.

## Inductive Bioscaffolds to Promote Brain Tissue Regeneration

The tissue repair response mediated by endogenous neurogenesis, however, does not lead to an invasion of progenitors into the lesion cavity to replace lost tissue. The tissue boundary is undergoing an injury response that seals off the cavity and aims to preserve viable tissue from ill effects of liquefactive necrosis. Unlike in wound healing, no substrate, such as a granulation tissue, is available in the cavity that can sustain the migration of brain cells to repopulate tissue. Indeed, this is evident in the peri-infarct area, where the repair response is using the available tissue substrate for migration and guides cells into position. A physical substrate to sustain cells within the cavity is therefore needed. Akin to fetal tissue transplants, experimentally this principle was demonstrated by implanting NSCs on scaffolds into a tissue cavity formed by a stroke. Provision of a structural support afforded a primitive *de novo* tissue formation by NSCs (Park et al., 2002; Bible et al., 2009, 2012a), although vascularization of this tissue was an issue (Bible et al., 2012b). It was also noted that large degradable solid microparticles produced a spiderweb-like tissue (Bible et al., 2009), hence producing an unfavorable condition to produce a homogenous tissue that integrates with the host. In contrast, implantation of NSCs in a hydrogel made out of ECM from decellularized urinary bladder matrix (UBM) and brain tissue resulted in an excellent cell survival in the tissue cavity, while producing a homogenous tissue that was distinct from host brain tissue (Bible et al., 2012a). These experiments indicated that a hydrogel formulation of a bioscaffold is favorable for replacing brain tissue. The hydrogel bioscaffold readily conforms to the cavity topology if a magnetic resonance imaging (MRI) guided injection-drainage approach is adopted (Massensini et al., 2015).

ECM scaffolds are widely used in peripheral soft tissue repair, including bladder (Santucci and Barber, 2005; Pearlman et al., 2018), dermis (Tchanque-Fossuo et al., 2017; Jeon and Kim, 2018), muscle (Sicari et al., 2014; Zhao and Bass, 2018), heart (Badylak et al., 2006; Khalil et al., 2018), and peripheral nerve (Nectow et al., 2012; Prest et al., 2018). These biomaterials can be sourced from different organs, such as the UBM (Freytes et al., 2008; Crapo et al., 2012, 2014; Dziki et al., 2017; Faust et al., 2017), umbilical cord (Koci et al., 2017), peripheral nervous system (Prest et al., 2018), spinal cord (Tukmachev et al., 2016), as well as the brain (Crapo et al., 2012; Medberry et al., 2013; Faust et al., 2017). It is important to note that the bioscaffolds are natural products that contain soluble (e.g., VEGF-A, BDNF) and juxtracrine factors (e.g., vitronectin, laminin), as well as structural proteins (e.g., collagen, hyaluronic acid) that can affect cellular functions (Saldin et al., 2017). The relative composition is dependent on the organ of origin, rather than being specifically designed, as in the case of synthetic materials (Wolf et al., 2015). Sheets of ECM are often used in peripheral tissue regeneration, but a minimally invasive implantable approach through narrow needles is desirable for brain applications, as this reduces damage to tissues overlying the cavity (Massensini et al., 2015). The formulation of ECM as a lyophilized digest that is reformulated as hydrogel serves this purpose (Freytes et al., 2008; Badylak et al., 2009) and affords reconstitution at different protein concentrations that determine bioscaffold stiffness (Massensini et al., 2015) and biodegradation (Ghuman et al., 2018).

Pre-gel ECM preparations are cytocompatible, while enhancing proliferation and migration of neural progenitors (Crapo et al., 2012; Crapo et al., 2014). Differentiation and neurite outgrowth of neural progenitors with UBM-ECM was higher compared to central nervous system (CNS)-derived ECM (Crapo et al., 2012; Faust et al., 2017), potentially demonstrating that non-CNS scaffolds might be favorable to induce tissue regeneration (Chan and Leong, 2008). In the spinal cord for instance, UBM-ECM performed as well as spinal cord-derived ECM, but provided favorable degradation kinetics (Tukmachev et al., 2016). Non-gelling UBM- and brain-ECM injections after traumatic brain injury revealed improvements in behavioral deficits, further highlighting their potential for therapeutic CNS applications (Zhang et al., 2013; Wu et al., 2016), but no tissue regeneration was reported due to the low concentration and small volume of injectate in these studies. However, these materials might have created a more favorable environment in damaged tissues to promote the survival of newly generated endogenous neural progenitors. Sourcing of homologous CNS tissues also poses a challenge due its low yield (Faust et al., 2017). It is important to point out that these acellular bioscaffolds are used to induce a host regenerative response and not to replicate the existing ECM that is found in a target organ. Heterologous organ sources therefore potentially exert a greater pro-repair effect than CNS-derived ECM.

## Immune System Response to Implantation

**FIGURE 4:**
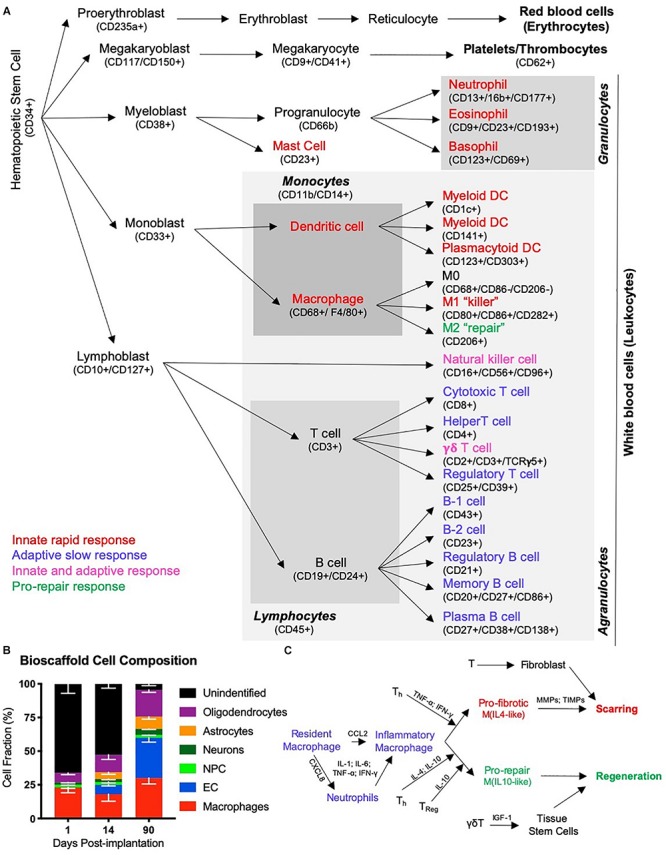
The immune response in tissue regeneration. **(A)** The inflammatory response is driven by the immune system. Although the immune system is a complex network of circulating and tissue-resident cells, these originate from a hematopoietic stem cell. Traditionally the immune system was characterized in studies of infection and cells have therefore been divided into those that contribute to an innate rapid response versus those that produce an adaptive slow response. Only a few types of cells, such as natural killer cells and γδT cells, have been thought to contribute to both. However, more recently the importance of inflammation in tissue repair and regeneration has revealed distinct phenotypic changes in cells, such as macrophages, that questions the traditional division into an innate and adaptive immune response to recognize a pro-repair response of the immune system. It is likely that a range of immune cells are involved in this pro-repair response, but that the function of cells might be different to their role in response to an infection. **(B)** A characterization of cells infiltrating an ECM bioscaffold implanted into a stroke cavity revealed that >75% of cells (mean – standard deviation) at 1 day post-implantation are not of a brain origin (Ghuman et al., 2018). Although the majority of these are currently unidentified phenotypes, it is likely that these are of an immune origin, such as neutrophils and eosinophils, which respond rapidly, but also transiently, to tissue changes. **(C)** Inflammation in wound healing is thought to be initiated by resident macrophages that release inflammatory cytokines (IL-1; IL-6, TNF-; IFN-) in response to detection of an injury. The chemokine CXCL8 is released and drives the rapid invasion of neutrophils into the damaged tissue from the blood. A secondary invasion response is recruiting inflammatory macrophages, as well as lymphocytes, such as helper (T_*h*_), regulatory (T_*reg*_) and γδT cells. T cells are thought to play a key role in modulating macrophage activity and promoting a pro-fibrotic response that results in tissue scarring or a pro-repair response that leads to tissue regeneration.

The initial inductive event after ECM implantation remains essentially unknown. It is conceivable that the physical implantation of a bioscaffold into a damaged tissue induces a pressure gradient that provokes a pro-inflammatory response from the immune system. Mechanotransduction in host tissue has also been associated with a good outcome in wound healing, but is generally neglected as an initiating event (Barnes et al., 2018). Invasion of cells needs to occur through existing tissue, as there are no blood vessels in the cavity that could support an invasion into the scaffold. Neutrophils and macrophages are dominant cell types acutely invading these scaffolds in peripheral tissues (Brown et al., 2009; Valentin et al., 2009), as well as the brain (Ghuman et al., 2016, 2018). However, cells resident within veterate tissue could provide the first response with low concentrations of ECM hydrogel permeating into damaged tissue (Massensini et al., 2015). The presence of juxtacrine and/or paracrine factors, as well as nanovesicles, released from the scaffold into tissue are potentially factors that induce an immune response in peri-cavity tissues (Sciari et al., 2014; Slivka et al., 2014; Dziki et al., 2017; Huleihel et al., 2017a). The acute invasion of immune cells is likely a reflection of innate immunity. This arm of the immune system involves neutrophils, eosinophils, basophils, dendritic cells, as well as macrophages (Figure 4A).

However, the acute immune response to inductive bioscaffolds in the brain remains poorly understood. The main focus has been on the role of macrophages, which constitute 23% of all cells found within ECM hydrogel implanted into the brain (Ghuman et al., 2016). Still, 66% of cells in the hydrogel at this time point have not been associated with a particular cell phenotype (Figure 4B) and could reflect other immune cells (i.e., neutrophils) contributing to a rapid innate response (Ghuman et al., 2018). It is unclear if macrophages found in the ECM hydrogel in the brain are derived from peripheral macrophages or if these are mostly brain-resident microglia that respond. More detailed studies of the mode of action (i.e., cellular level) involved in this early phase are required to gain a better understanding of the factors (i.e., mechanisms of action) that elicit the initial response and provoke a regenerative cascade (Badylak and Gilbert, 2008). Understanding this process is especially important for the design of synthetic bioscaffolds aiming to replicate the tissue regeneration achieved with natural materials (Drury and Mooney, 2003; Zhu and Marchant, 2011; Aurand et al., 2012).

Immune cell recruitment is most likely mediated through soluble factors that can diffuse through a large volume of tissue, although it is influenced by tissue density and other structures (e.g., scarring) that provide barriers (Nicholson, 2001). Notch and PI3K/Akt signaling in ECM bioscaffolds, for instance, have been associated with the phagocytic activity of immune cells (Slivka et al., 2014). The migration of macrophages is dependent on monocyte chemoattractant protein-1 (MCP-1/CCL2), which guides cells to their target (Deshmane et al., 2009). During the acute phase of macrophage invasion, most of these monocytes (>35%) exhibit an M1-like phenotype (CD86+), but by 14 days this proportion is reduced and mostly (>20%) M2-like (CD206+) characteristics are found (Ghuman et al., 2018). This shift in macrophage phenotype is also thought to affect the differentiation of organ-specific cells, such as neurons, in the case of the brain (Faust et al., 2017). In wound healing, a shift toward an M2-like phenotype is thought to be influenced by lymphocytes (i.e., T and B cells) that invade as part of the adaptive immune response (Figure 4C). IL-4 is commonly associated with this shift in macrophage phenotype, but juxtacrine factors, such as MEK/ERK and integrin signaling are also known to decrease phagocytic activity and promote a repair phenotype (Slivka et al., 2014). There is also recent evidence that ECM bioscaffolds produce a macrophage activation that might be distinct from the characteristics associated with M0, M1 or M2 activation (Huleihel et al., 2017b), but this might merely be a reflection of the activating signals rather than the cells’ function (Novak and Koh, 2013). Other classifications, such as division of macrophages into pro-fibrotic and pro-repair phenotypes have also been proposed to be more relevant to the processes involved in tissue repair and regeneration (Sadtler et al., 2019).

**FIGURE 5:**
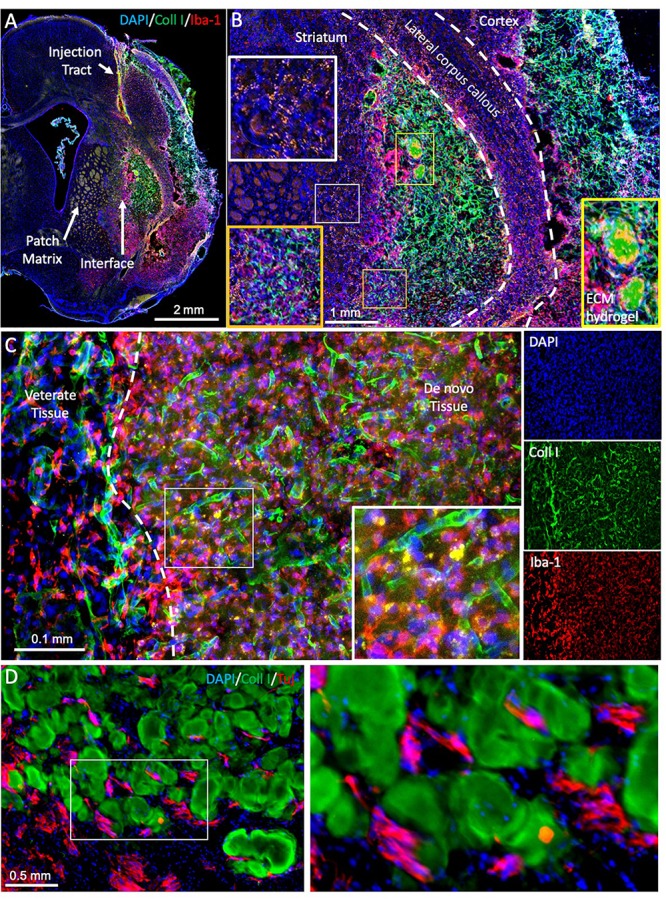
Evaluating brain tissue regeneration. **(A)** A bioscaffold is implanted into the lesion cavity through a narrow bore needle that produces an injection tract and defines the trajectory of the injection. The aim of the procedure is to fill the tissue cavity and to produce a close-fitting interface with host tissue that affords invasion of cells into the scaffold and produces a seamless integration between veterate and *de novo* brain tissue (Ghuman et al., 2018). *De novo* tissue growing inside the cavity can be identified based on collagen I staining of the ECM hydrogel and the neovasculature, surrounded by veterate tissue, as indicated by the peri-infarct area and a dense presence of microglia (Iba1 + cells). **(B)** At 90 days post-implantation, the bioscaffold is almost completely degraded. In this case, two small remnants of the ECM hydrogel, characterized by a dense collagen I content are still undergoing cell invasion and degradation (yellow box). However, the rest of the scaffold is degraded and replaced with *de novo* tissue, in which blood vessels contain a higher level of collagen I compared to host tissue. Regenerated tissue (orange box) has blood vessels high in collagen I, but a dense tissue structure is evident with a reduced number of Iba-1 positive microglia/macrophages. It was also noted that in *de novo* tissue, some particulates were present that were not evident in veterate brain (white box). **(C)** In regenerating tissue, the bioscaffold is degraded, but there is still a higher collagen I background compared to host brain. Morphologically it is also distinct with a higher content of microglia/macrophages and strongly collagen I positive blood vessels. Nevertheless, more robust unique identifiable markers are desirable to contrast these different microenvironments. **(D)** In between patches of ECM bioscaffold, tissue is developing that contains neurons (Tuj) at a higher density than within the bioscaffold. However, at present there are no robust markers that allow a reliable identification of this as *de novo* tissue, complicating the quantification of the regenerative process.

Although macrophage activity within ECM bioscaffolds has been extensively investigated, their interaction with other cells remains poorly understood, especially in the context of an adaptive (delayed) immune response (Figure 5A), which has also been reported to participate in tissue regeneration (Strbo et al., 2014; Sadtler et al., 2019). The use of xenogenic matrix grafts raises the question of the role of an adaptive immune response to foreign proteins. The blood-brain barrier (BBB) produces an immune-privileged tissue environment, but drainage of solutes through the glymphatic system leads to a sensitization of B and T cells in the cervical lymph nodes (Verheggen et al., 2018). There is evidence that T helper cells respond to xenogenic proteins in ECM bioscaffolds, suggesting that a specific immune response develops (Allman et al., 2001). Still, it remains unclear if an “adaptive immune response” is an indication of an incomplete decellularization, which might lead to remnants of cells being retained with the bioscaffold to produce a sensitization of the immune system. It is generally thought that ECM molecules by themselves do not elicit an adaptive immune response. Still, the complexity of molecules contained within decellularized materials (e.g., microvesicular bodies) can provide a range of targets for an adaptive immune response that is distinct from the ECM molecules *per se*. An adaptive immune response to even endogenous proteins can follow an innate immune response, as in the case of stroke for instance (Rayasam et al., 2018). The division between an innate and adaptive immune response is based on infection studies and might therefore not be an adequate description of the response observed after tissue damage and repair (Sadtler et al., 2019). A greater focus on the role of the immune system in these conditions is likely to provide a more adequate understanding and conceptualization of the role inflammation plays in modulating a tissue response toward repair and regeneration.

## Biodegradation of Scaffolds

The recognition that the same immune cells can exert different functions is part of this evolving conceptualization. For instance, the polarization toward an M2-like phenotype is thought to be crucial for the repair response, even though M1-like macrophages persist for 90 days and are thought to play an essential role in the degradation of the bioscaffold (Ghuman et al., 2018). Ablation of peripheral macrophages prevents structural remodeling of ECM bioscaffolds, further highlighting their crucial role in this process (Valentin et al., 2009). Macrophages secrete proteases that degrade cross-linked collagen scaffolds (Yahyouche et al., 2011; Madsen et al., 2013), as is the case with ECM hydrogel. Especially, MMP-2 and MMP-9 (Table 2) are upregulated after acute brain injuries, such as stroke, by secretion from brain microglia reacting to the tissue insult (del Zoppo et al., 2007), but so are MMP-3, MMP-7, MMP-10, and MMP-11 (Rempe et al., 2016). UBM-ECM hydrogel is especially high in collagen I (Saldin et al., 2017), which is primarily degraded by MMP-1, MMP-2, MMP-8, MMP-12, and MMP-13 (Webster and Crowe, 2006; Lu et al., 2011). In contrast, MMP-9 is mostly associated with degradation of the basement membrane around blood vessels, which is rich in collagen IV, laminin, and vitronectin (Fukuda et al., 2004; Underly et al., 2017). Neutrophils also produce MMP-9 and are known to be involved in ischemic tissue regeneration in peripheral limbs (Heissig et al., 2010). Other proteases, such as A Disintegrin And Metalloproteinases (ADAMs) and Meprins, are also likely involved in ECM remodeling, but their role remains poorly understood, especially in the context of tissue regeneration (Bonnans et al., 2014). How different immune cells and their activation state drive protease production and bioscaffold degradation also requires more mechanistic studies to understand their role in initiating the tissue regeneration process.

**TABLE 2:** Matrix metalloproteinases (MMPs).

**Family Member**	**Aliases**	**Activators**	**Collagen Targets**												**Other ECM Targets**
			**I**	**II**	**III**	**IV**	**V**	**VI**	**VII**	**VIII**	**IX**	**X**	**XI**	**XIV**	
MMP-1 (1) secreted	Collagenase-I Interstitial Collagenase	MMP-3 MMP-10 Plasmin Kallikrein Chymase	^∗^	^∗^	^∗^			^∗^	^∗^			^∗^			Aggrecan Entactin Gelatin Perlecan Tenascin
*MMP-2 (2) secreted*	Gelatinase A	MMP-1 MMP-7 MMP-13 MMP-14 MMP-15 MMP-16 MMP-24 MMP-25 Plasmin	^∗^		^∗^	^∗^	^∗^		^∗^			^∗^	^∗^	^∗^	Aggrecan Elastin Fibronectin Gelatin Laminin
*MMP-3 (1) secreted*	Stromelysin-I	Plasmin Kallikrein Chymase Tryptase			^∗^	^∗^					^∗^	^∗^	^∗^		Aggrecan Decorin Fibronectin Gelatin Laminin Perlecan Tenascin
*MMP-7 (3) secreted*	Matrilysin-1 PUMP-1	MMP-3 MMP-10 Plasmin				^∗^						^∗^			Aggrecan Decorin Elastin Fibronectin Gelatin Laminin
MMP-8 (1) *secreted*	Collagenase-2 Neutrophil collagenase	MMP-3 MMP-10 Plasmin	^∗^	^∗^	^∗^		^∗^		^∗^	^∗^		^∗^			Aggrecan Gelatin
*MMP-9 (2) secreted*	Gelatinase B	MMP-2 MMP-3 MMP-10 MMP-13 Plasmin				^∗^	^∗^		^∗^			^∗^		^∗^	Aggrecan Decorin Elastin Gelatin Fibrin Laminin Vitronectin
*MMP-10 (1) secreted*	Stromelysin-2	Plasmin Kallikrein Chymase Tryptase			^∗^	^∗^	^∗^		^∗^		^∗^	^∗^			Aggrecan Fibronectin Laminin
*MMP-11 (1) secreted*	Stromelysin-3	Furin Plasmin													Aggrecan Fibronectin Gelatin Laminin
MMP-12 (1) *secreted*	Macrophage Metalloelastase	n.d.	^∗^			^∗^									Aggrecan Elastin Fibronectin Laminin Nidogen Osteonectin
MMP-13 (1) *secreted*	Collagenase-3	MMP-2 MMP-14 Plasmin Kallikrein Chymase Tryptase	^∗^	^∗^	^∗^	^∗^			^∗^		^∗^	^∗^		^∗^	Aggrecan Fibronectin Laminin Osteonectin Perlecan Tenascin
MMP-14 (4) membrane	MT1-MMP	Furin	^∗^	^∗^	^∗^										Aggrecan Fibrin Fibronectin Gelatin Laminin
MMP-15 (4) membrane	MT2-MMP	Furin													Aggrecan Fibronectin Laminin Nidogen Perlecan Tenascin
MMP-16 (4) membrane	MT3-MMP	Furin			^∗^										Fibronectin Gelatin
MMP-17 (4) membrane	MT4-MMP	Furin													Fibrinogen Gelatin
MMP-18	Collagenase-4 XCol4 Xenopus Collagenase	n.d.	^∗^												n.d.
MMP-19	Stromelysin-4 RASI-I	n.d.	^∗^			^∗^									Fibronectin Gelatin Laminin
MMP-20 secreted	Enamelysin	n.d.													Aggrecan
MMP-21 (1) secreted	XMMP	n.d.													n.d.
MMP-23 membrane	CA-MMP	n.d.													Gelatin
MMP-24 (4) membrane	MT5-MMP	n.d.													Fibrin Gelatin
MMP-25 (4) membrane	MT6-MMP	n.d.				^∗^									Gelatin Fibrin Fibronectin Laminin
MMP-26 (3)	Matrylisin-2 Endometase	n.d.				^∗^									Gelatin Fibronectin
MMP-27 (1)	MMP-22 Chick embryo-MMP	n.d.													Gelatin
MMP-28 secreted	Epilysin	n.d.													n.d.

*MMPs can be divided into several sub-domains consisting of MMPs with basic domains (i.e., prodomain + catalytic domain + hemopexin-containing ancillary domain) (1); MMPs with minimal domains (i.e., prodomain + catalytic domain) (3), MMPs with fibronectin-domain inserts (2), Membrane-bound MMP anchored by GPI or a transmembrane domain (4). There is a further distinction in MMPs either being secreted or membrane-associated. A variety of aliases have been used to refer to different MMPs, depending on their original discovery. MMPs typically also act as activators of other MMPs, further enhancing their potential to degrade different types of collagen, as well as other types of ECM molecules. A few MMPs have been associated with acute brain injury, as indicated by *italics*, but other have been found to be upregulated in different types of neurodegenerative disease, as well as synaptic plasticity. MMPs therefore serve important roles in maintaining a healthy brain environment, as well as being propagators of tissue damage in disease conditions (n.d., not defined).*

Although collagen I is considered a major structural target for biodegradation, the role of the biodegradation of other ECM molecules remains poorly understood. In ECM hydrogels implanted into the brain, for instance, fibronectin and chondroitin sulfate were mostly degraded within a week, even though collagen I was still abundantly present (Jin et al., 2017). Laminin, collagen IV and hyaluronic acid (HA) were also present at 1 day post-implantation (Massensini et al., 2015), but it remains unclear if these molecules are rapidly degraded or if the degradation profile of the ECM molecules is equivalent to collagen I. Less abundant molecules could be degraded faster than more abundant one’s, even though the rate of biodegradation is the same. A quantitative comparison between different ECM molecules and their degradation is needed to address these key questions. While bioscaffolds from different sources are thought to exert similar regenerative effects (Keane and Badylak, 2015; Tukmachev et al., 2016), their biodegradation is likely to be influenced by their composition. Understanding how individual ECM molecules are degraded and lead to the release of other factors (e.g., VEGF-A) will be essential to design novel biomaterials that afford a greater control over biodegradation (Sridharan et al., 2015; Kelly et al., 2017).

Biodegradation of ECM bioscaffolds in the brain potentially follows a different dynamic than in peripheral organs. Stiffer ECM hydrogel (8 mg/mL) undergoes a slow degradation in the brain, with only a 32% reduction in volume by 90 days, whereas softer bioscaffolds (3 and 4 mg/mL) were almost completely absorbed (>95%) (Ghuman et al., 2017, 2018). Stiffer brain-compliant 8 mg/mL UBM-ECM hydrogel was rapidly invaded by macrophages compared to the weaker gels, but the density of cells inside the scaffold decreased over time indicating that a poor biodegradation occurred (Ghuman et al., 2018). Softer gels produced a more consistent cell density (∼4000 cells/μL) with a vasculature forming, which was absent in stiffer material. Although these differences indicate a potential mode of action for biodegradation, it remains unclear how stiffness or the higher protein concentration adversely affected biodegradation, considering that there was an extensive cellular response at 1-day post-implantation in the bioscaffold that did not readily degrade (Ghuman et al., 2016). In peripheral tissues, a complete replacement with host tissue between 75 and 90 days has been associated with tissue regeneration (Record et al., 2001; Gilbert et al., 2007; Valentin et al., 2009; Carey et al., 2014; Dearth et al., 2016) and is consistent with the time course observed with softer gels in the brain (Ghuman et al., 2018). However, in peripheral tissues most biodegradation studies investigate the use of ECM sheets, rather than hydrogel. It is therefore unclear if ECM hydrogel will follow a similar time course in peripheral tissues. Crucially, the lack of biodegradation in the brain did not lead to tissue regeneration (Ghuman et al., 2017), whereas biodegradation of the scaffold produced *de novo* tissue (Ghuman et al., 2018). Biodegradation gradually removed the bioscaffold and provided the structural basis for new cells to invade the cavity. These newly invading cells showed assembly in and inbetween the ECM hydrogel to initiate the formation of new tissue. This process is very similar to that described in peripheral tissues, where biodegradation or the remodeling of a “transient” ECM is a key step to replace lost tissues (Swinehart and Badylak, 2016).

## Neural Tissue Formation and Connectivity

Tissue-specificity is determined by the cells that invade from the host organ into the degrading bioscaffold. The continued proliferation and response of the SVZ to brain injury (Kazanis et al., 2013; Chang et al., 2016) provides a ready supply of local (i.e., peri-cavity) neural progenitors that can respond to the implantation of a bioscaffold. Inflammatory factors released from microglia and invading immune cells provide a trail of ligands, such as CCL2 (a.k.a. monocyte chemoattractant protein 1) and CXCL12 (a.k.a. stromal-derived factor-1α), for neural progenitors to follow (Bye et al., 2012; Kaneko et al., 2017). Some local proliferation might also occur in response to factors, such as bFGF (FGF2) and VEGF-A, released by activated astrocytes (Gabel et al., 2016). Release of these factors can occur from the ECM bioscaffold and lead to the initial cell invasion of neural progenitors and astrocytes observed within 1 day in implanted gels (Ghuman et al., 2016). However, the continued recruitment of neural progenitors from veterate brain is likely dependent on the secretion of appropriate factors, such as CCL2 and CXCL12, from microglia and/or macrophages that are degrading the scaffold. It can be surmised that in the case of the 8 mg/mL ECM hydrogel implanted into a stroke, the very slow biodegradation associated with very few macrophages present in the scaffold provided insufficient chemokines to drive host cell invasion, including blood vessels (Ghuman et al., 2018). Blood vessels are likely to play a triple role. Their formation in the scaffold affords a direct route for macrophage invasion to accelerate biodegradation, but they also provide a migration substrate for neural progenitors and are required to meet the metabolic demands of a newly forming tissue (Serbo and Gerecht, 2013).

The cellular composition inside the bioscaffold shifts from predominantly immune cells, such as macrophages, being present to endothelial cells, as well as neural cells, such as oligodendrocytes, neurons, neural progenitor cells, and astrocytes. These constitute over 70% of cells at 90 days (Figure 4B) (Ghuman et al., 2018). This suggests that some cellular composition required for *de novo* tissue formation precedes the degradation of the implanted ECM. This is consistent with new ECM being deposited by site-appropriate cells, rather than immune cells. Still, the density of neurons (6% of cells) inside the hydrogel is considerably lower at 90 days compared to intact tissue (42%) (Ghuman et al., 2018). This is also the case during development, where radial glia provide the substrate for neuron progenitors to migrate into position and gradually produce the neuronal density of an adult brain (Malatesta and Gotz, 2013). Astrocytes present within the bioscaffold, however, do not morphologically or topologically resemble radial glia (Ghuman et al., 2016). The transitional microenvironment in the bioscaffold, where neural cells are found, does not contain strands of radial glia along which neural progenitors migrate.

Regenerating tissue produces unique microenvironments not present in development. Notably, intact brain tissue borders on peri-cavity damaged tissue, with this damaged tissue transitioning into *de novo* tissue (Figure 5A). This *de novo* tissue borders in turn on the bioscaffold still undergoing constructive remodeling. The cytoarchitecture within the bioscaffold does not reflect the density of *de novo* or damaged tissue (Figure 5B). Identification of neural cells inside the bioscaffold is facilitated by visualizing the ECM hydrogel, traces of which often remain even at a low concentration (Figure 5C). However, a definition of *de novo* tissue, which can develop in between patches of degrading bioscaffolds (Figure 5D), is more challenging. Its cytoarchitecture and composition therefore currently remains unknown. To contrast *de novo* from damaged peri-cavity veterate tissue is challenging, as there are no specific markers for these microenvironments. Positional specification markers might provide some guidance to identify the differentiation of site-appropriate cells, but these are likely to be expressed inside the bioscaffold, damaged and *de novo* tissue. Deposition of ECM molecules might have greater potential to distinguish these different microenvironments. For instance, the formation of new blood vessels follows a sequential deposition of vitronectin, fibronectin, laminin, collagen I and collagen IV (Chou and Modo, 2016). Establishing a similar deposition pattern for the interstitial ECM could lead to a better characterization of microenvironments. The use of specific tags for the bioscaffold would further afford a distinction from veterate tissue that does not rely on the abundancy of ECM molecules in the hydrogel (Park et al., 2019).

Akin to the transplantation of fetal tissue, the integration between newly forming and veterate tissue will be a key aspect to produce behavioral recovery that is dependent on neuronal circuitry. Axons containing neurofilament are found within ECM hydrogel implanted in a stroke, as well as in *de novo* tissue forming in between patches of remaining scaffold (Ghuman et al., 2018). Oligodendrocytes were the most abundant neural cell phenotype within the ECM hydrogel, but it remains unclear if these myelinate axons. Implantation of angiogenic HA hydrogel indicated axonal regeneration through a cortical cavity along newly forming blood vessels (Nih et al., 2018). Wider connectivity with veterate tissue or the formation of neuronal circuitry in *de novo* forming tissue remain to be investigated. Extensive work on axon regeneration has been focused on the spinal cord (Anderson et al., 2018), but a more complex topology of connections is required to establish neuronal circuitries in the brain, as well as to establish a functional integration between *de novo* and veterate tissue. Although fetal tissue transplants and implantation of HA hydrogel indicate that new axonal connections can be formed in or through cavities, it is unclear if guidance to newly forming connections is required to ensure the appropriate development of neuronal circuitry. So far, no epileptic fits have been reported, potentially suggesting that spontaneously appropriate connections are formed or that there is a lack of functional integration (Modo and Badylak, 2019). The use of conductive hydrogels and specific stimulation might provide a new perspective to promote an integration of newly forming tissues (Oh and George, 2019). The time course of establishing axonal connections and their potential underpinning of behavioral functions remain to be investigated, but it is expected that neuronal differentiation in *de novo* tissue formation precedes axonogenesis.

## Cell-Bioscaffolds and Engineered Micro-Tissue Constructs for Repair and Regeneration

Endogenous tissue regeneration is preferable, as it will use the patients’ own cells and avoid potential immune rejection. However, endogenous tissue regeneration is a lengthy process, potentially taking over 1 year to complete. Moreover, the reservoir of neural progenitors in the brain might be insufficient to supply sufficient neurons to repopulate a large *de novo* tissue. Alternative engineering strategies therefore need to be considered to expand the neural substrate required for tissue restoration (Chen et al., 2016). Implantation of allogenic neural cells in a biomaterial can potentially increase the neural substrate required to restore lost tissue. Most studies to date have focused on the use of biomaterials, such as hydrogels, to improve the delivery of cells to the brain (Ho et al., 2019). Only a few studies have aimed at restoring tissue inside the cavity. Bioscaffolds combined with neural stem cells will provide the structural support to retain cells in the cavity and allow them to differentiate (Park et al., 2002; Bible et al., 2009, 2012a). Implantation of fetal-derived neural stem cells has the potential to produce a cell substrate that is positionally specified to be striatal or cortical tissue (Bible et al., 2012a,b), but so far no evidence for the generation of region-specific tissue has been reported. Induced pluripotent stem cells (iPS) will require positional specification *ex vivo* or *in vivo*. Implantation of NSCs requires the invasion of host endothelial cells to form a vasculature, as well as microglia to provide their support function. NSCs implanted with a non-angiogenic bioscaffold do not facilitate a neovascularization (Bible et al., 2009) and require additional instructive signals, such as release of VEGF-A, to ensure a re-vascularization (Bible et al., 2012b). Moreover, implantation of NSCs can exert an anti-inflammatory effect that prevents or reduces the infiltration of microglia. In the case of inductive bioscaffolds, this could lead to a reduction in bioscaffold degradation (Bible et al., 2012a). The inductive properties of the bioscaffold are, however, less important in this approach. In contrast to endogenous brain regeneration, implanted cells should provide a rapid replacement of tissue and hence a site-appropriate ECM might be more important. It is noteworthy that neural stem cells in brain ECM accelerate the formation of neuronal circuitry (Lam et al., 2019).

A rapid tissue restoration might therefore require the key “ingredients” for brain tissue, notably NSCs, ECs and ECM. The ratio between NSCs and ECs is crucial to promote the formation of a vasculature *in vitro* (Chou et al., 2014; Chou and Modo, 2016), as well as *in vivo* after implantation into a stroke cavity (Nicholls et al., 2015). ECs are highly immunogenic and upon formation of a vasculature in *de novo* tissue will lead to exposure of the endothelial wall to circulating immune cells, as in the case of fetal tissue or whole organ transplants. This will require a continued immunosuppression that potentially compromises the quality of life of patients. However, it is not expected that immunosuppression, such as cyclosporin A, will affect the regenerative response *per se*, as these pharmacological agents affect the indirect chronic immune response mediated through lymphocyte stimulation and not microglia/macrophages (Karam and Wali, 2015). The low immunogenicity of NSCs does not raise this issue, with good survival in the absence of immunosuppression (Modo et al., 2002, 2003). In contrast, implantation of microglia could potentially cause greater concerns, as these are highly immunogenic, and could mount a graft-versus-host response (Nassereddine et al., 2017), where implanted microglia would attack host cells. Cellular sourcing hence will be an issue with the co-implantation of cells that are immunogenic. iPS cells can circumvent these issues, but the protracted time to grow sufficient cells *ex vivo* would be a concern that would need to be balanced against the time course of endogenously induced tissue regeneration.

Beyond the co-implantation of NSCs and ECs to “spontaneously” form a tissue *in situ*, the *ex vivo* construction of micro-tissues is also considered a potential therapeutic intervention aimed at replacing lost tissue. Stem cell niches can be engineered for implantation to provide a continuous supply of new cells to repair or reconstruct brain tissue (Lampe and Heilshorn, 2012). Preformed neurovascular units can also be envisaged for implantation (Potjewyd et al., 2018). *Ex vivo* growing of brain organoids for transplantation can potentially accelerate *in vivo* tissue growth, as an essential cytoarchitecture and rudimentary connectivity can be pre-established (Mansour et al., 2018). Larger constructs with pre-formed axonal projection can also be envisaged (Struzyna et al., 2015, 2017) and in some cases be designed to specifically replace a major fiber tract, such as the nigrostriatal pathway, which is almost completely lost in Parkinson’s disease (Struzyna et al., 2018). Pre-assembly of micro-tissue is potentially an exciting approach to pre-define an cytoarchitecture using bio-printing to produce homologous constructs more rapidly than spontaneously forming tissue (de la Vega et al., 2019). Delivery of these complex constructs will, nevertheless, be more challenging through a narrow bore needle without deforming or destroying the construct (Wahlberg et al., 2018). Creating a tight interface with veterate tissue also needs considering to ensure integration, as the topology is very different between subjects. Preformed connections could adversely affect cell viability, if these connections are cut or damaged during the implantation process, similar to fetal tissue transplants in Parkinson’s disease (Schierle et al., 1999). Implantation of tissue constructs is therefore likely to be a more complex procedure to achieve, especially in terms of intracerebral delivery and cell sourcing.

## Training of Functional Circuitry

Axonal projections lead to connectivity between regions, but synaptic connectivity is required to avoid synaptic pruning and a potential die-back of axons. To avoid synapse pruning, functional synapses need to be established between axons and dendrites (Butz et al., 2009). Synaptogenesis will establish connections, but it is their activation through a common input that renders these functionally dependent and avoids pruning. These so called Hebbian synapses underpin plasticity in the damaged brain, which is exploited during rehabilitation training (Dalise et al., 2014; Takeuchi and Izumi, 2015). Transplantation of NSCs and their functional integration into neuronal circuits is also dependent on synaptic integration between grafted and host cells (Tornero et al., 2013, 2017). However, mapping these connections across a whole brain poses a formidable challenge (Doerr et al., 2017). Although day-to-day activities lead to some functional recovery, this is further enhanced by providing an enriched environment to animals to increase activity and improve the establishment of functional synapses. An enriched environment leads to diverse changes in function, as well as anatomical markers of grafted cells (Dobrossy and Dunnett, 2001). However, introduction of a rehabilitation paradigm does not always lead to an improvement in graft-mediated recovery (Hicks et al., 2009). Only a few studies have investigated the interaction between rehabilitation after intra-striatal grafting of fetal tissue into striatal lesions modeling Huntington’s disease (Dobrossy and Dunnett, 2004, 2008) or stem cells after a stroke (Hicks et al., 2007, 2008, 2009). Although the effects of rehabilitation on synapse formation in transplanted cells is potentially profound, our current state of knowledge about these interactions is unsatisfactory and requires more extensive studies that define appropriate rehabilitation paradigms and how these differ from interventions without cell therapy (Ghuman et al., 2018). Establishing optimal intervention periods for when these programs should be administered, as well as how long a rehabilitation program is needed in conjunction with “regenerative therapies” to achieve a maximal integration requires detailed investigations to ensure clinical efficacy (Savitz et al., 2014; Ross et al., 2016).

The role of training new tissue in the context of brain tissue regeneration remains unaddressed. However, there is evidence from ECM bioscaffolds regenerating muscle tissue that rehabilitation training is an essential element to produce functional tissue (Gentile et al., 2014; Sicari et al., 2014). Rehabilitation tasks are likely to be dependent on behavioral deficits after brain injury, as these are dependent on the regional location of damage (Feys et al., 2000). The rehabilitation paradigm will therefore need to adapt depending on the functional deficit (Kreber and Griesbach, 2016), which will complicate group-wise comparisons as no one rehabilitation paradigm will be suitable for all subjects (Gentile et al., 2014). In the case of damaged tissue, neuronal progenitors from the SVZ will integrate and supplement existing neuronal networks to improve their function. In contrast with tissue regeneration, a complete new set of neuronal networks is created that needs to form a functional circuit based on a behavioral input. Tissue replacement is expected to restore function of lost tissue, but akin to learning in development it can be expected that new tissue needs to be trained to function appropriately (Dobrossy and Dunnett, 2001; Dobrossy and Nikkhah, 2012). In conventional rehabilitation, plasticity of existing networks can be induced by exercise (Dobkin, 2008), which leads to the release of growth factors, such as BDNF, that support synaptogenesis (Dalise et al., 2017). To ensure a functional synapses formation, task integration is required (Rensink et al., 2009). In animal models, behavioral testing can be considered a form of task integration. However, ideally a separate task will be used to integrate and evaluate functional effects (Ghuman et al., 2018). Compensation (i.e., solving the same task using an alternative strategy) might occur, which will confound the assessment of efficacy. Extensive testing on a particular behavioral task could increase compensation effects, as the animal is learning novel ways to solve the task (Boltze et al., 2014). Separating training and testing should therefore be considered the gold standard for a behavioral evaluation of tissue regeneration and the use of rehabilitation to train *de novo* tissues.

## Criteria to Evaluate Functional Brain Tissue Regeneration

The evaluation of behavioral effects of brain tissue regeneration needs to be performed in contrast to subjects that only experienced brain injury, as some spontaneous recovery of deficits occurs. Learning and compensatory strategies could be adopted to improve performance that would not reflect a therapeutic benefit. Inclusion of appropriate non-injured controls is required to establish a performance baseline over time, as well as an effect size for the deficit and potential recovery. Considering the invasiveness of the procedure to deliver a bioscaffold, appropriate surgical controls (e.g., needle implantation, vehicle injection) need to be evaluated, but only need to be included consistently in studies if there are significant behavioral effects. Eventually, more detailed studies are required to determine the influence of biological variables (e.g., age, sex) on brain tissue regeneration. Specifically, subject age is known to affect inflammation, ECM composition and its potential to induce a regenerative response (Brown et al., 2017; Hachim et al., 2017; LoPresti and Brown, 2018). Inflammation is another biological variable that is known to be affected by age and sex, but little is known about regenerative differences between sexes. To gain a robust mechanistic understanding, effects of all these variables need to be accounted for.

Although improvements in behavioral deficits are the ultimate aim of this approach, it cannot be expected that initial studies defining the important variables to achieve tissue regeneration will reveal behavioral improvements. Initial studies should aim to achieve a robust and reproducible tissue formation by defining key rules, such as the formation of a tissue cavity prior to bioscaffold implantation etc. (Table 3). Histological analyses need to establish robust methods that can contrast damaged tissue from regenerating tissue. Particular ECM molecules might provide differential markers for this purpose, but need to be validated. In the absence of this validation, it is possible that mechanistic interventions will target damaged veterate brain, rather than regenerated brain. This would lead to erroneous interpretations of functions in regenerated brain tissue.

**TABLE 3:** Emerging rules for an induced brain tissue regeneration.

**Rules**	**Description**
1	The adult mammalian brain does not spontaneously restore lost tissue.
2	Preserved neurogenic regions are required to produce endogenous neurons.
3	Distance of neurogenic regions to areas of damage is crucial to induce a response.
4	Extent of cellular reservoir in neurogenic regions is crucial to produce sufficient cells to promote repair and regeneration.
5	Neurogenic regions need to produce cells with site-appropriate positional specification.
6	A tissue substrate needs to be introduced to provide support for cell invasion.
7	A continuous interface between scaffold and tissue is required to ensure a homogenous tissue integration.
8	Formation of a mature glial scar prior to introducing a tissue substrate will complicate tissue regeneration and integration.
9	A rapid invasion of cells is required to ensure a regenerative cascade is initiated.
10	The tissue substrate needs to be degraded to >90% within 3–4 weeks to allow *de novo* tissue to form.

A distinction between behavioral and functional effects is made to differentiate overt observations related to performing a specific task (i.e., behavior) from brain activity and tissue metabolism (i.e., function). We further propose that the claim of establishing a *de novo* functional brain tissue should be provided by non-invasive imaging techniques, such as MRI. A pre-implant image of the tissue cavity and the host brain should be provided with further images charting how the cavity is transformed by tissue regeneration (Ghuman et al., 2017). This *in vivo* time-lapse in combination with histology should be the gold standard to accept claims of tissue restoration. The cytoarchitectural organization of *de novo* tissue should be contrasted with intact brain tissue, as well as the phenotypes of cells within the bioscaffold. These two microenvironments provide benchmarks that contrast regenerating and target tissue. Proof of tissue dynamics and a restorative process occurring over time are required to substantiate the processes involved in brain tissue regeneration, eventually leading to mode and mechanism of action studies.

To this end, we further suggest that proof of the bioscaffold’s presence, retention, distribution and degradation are required to indicate their pivotal role in tissue regeneration. Although a reliance on morphological and protein concentration difference can serve at early time points post-implantation to identify the bioscaffold (Massensini et al., 2015), as these materials are degraded, protein concentrations are increasingly similar to veterate brain and regenerating tissue is morphologically increasingly similar to damaged or intact brain tissue (Ghuman et al., 2016; Jin et al., 2017). Ideally, unique identification strategies for bioscaffolds are implemented (e.g., labeling) without affecting the biological activity of the material (Park et al., 2019). This should allow the identification of even small quantities of bioscaffold that might still undergo degradation or be incorporated into the new tissue. *In vivo* monitoring of the bioscaffold (Liang et al., 2015; Jin et al., 2017; Piejko et al., 2019), as well as cells (Nicholls et al., 2015, 2016) would further improve our understanding of how biomaterials and cells interact to form an integrated brain tissue.

**FIGURE 6:**
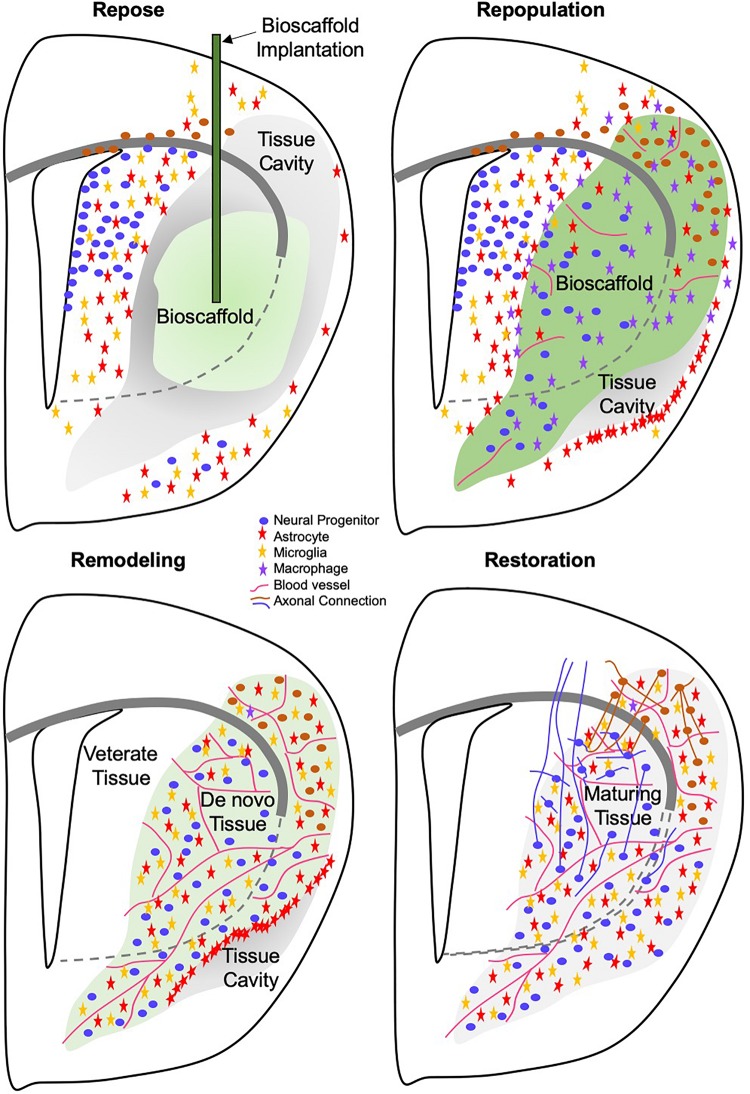
Four phase of brain tissue regeneration. Phase 1: The repose phase is characterized by tissue loss being mostly complete and a repair response having been instigated. Although gliosis is ongoing, no defining scar along the tissue cavitation has emerged. During this phase, a bioscaffold can be implanted to initiate the tissue regeneration process. In the absence of an introduction of a bioscaffold a scar is forming around the cavity. Phase 2: During the repopulation phase, host cells are invading the bioscaffold. Even during the acute invasion phase, brain derived cells, such as neural progenitors and astrocytes, are infiltrating the biomaterial, but immune cells are more rapidly invading and provide additional soluble and juxtracrine signaling to recruit host brain cells to repopulate the tissue cavity. Phase 3: Invading cells deposit transient matrix molecules and take-up positions inside the bioscaffold that leads to a gradual degradation of the scaffold. Blood vessel formation during this phase plays a key role to promote biodegradation, but also to remodel individual compartments that will develop into neuropil. Within the vascular compartment, cells are depositing appropriate matrix molecules, such as vitronectin, laminin and collagen. In the neuropil, neural cells deposit matrix molecules, such as laminin, aggrecan, decorin, thrombospondin that are involved in maintaining structure and juxtracrine signaling. At the end of this phase, the bioscaffold is completed degraded and replaced with host matrix. Phase 2 and Phase 3 overlap within different parts of the cavity. Phase 4: Once host brain cells are in position and formed a neuropil in between blood vessels, tissue maturation is occurring with terminal differentiation of neurons through interaction with astrocytes, oligodendrocytes and ECM molecules. It can be anticipated that *de novo* tissue formation in phase 3 and maturation processes, such as axonal and dendritic branching, can occur side-by-side. Axonal and dendritic processes are required to form a functional neuronal circuitry.

Finally, it is important to develop a framework that will define the sequence of cellular dynamics (e.g., neutrophils invade first and facilitate macrophage recruitment) that drive different phases of brain tissue regeneration. Are these events dependent on each other? What happens if we interfere with individual processes? Does this lead to a disruption of the regenerative cascade or does it lead to a different type of tissue? In contrast to wound healing, which mainly focuses on reintegrating adjoining tissues, we here propose that (1) brain tissue needs to repose (i.e., cavitation needs to be mostly complete without maturation of a surrounding glial scar) to receive an inductive bioscaffold; (2) the lack of scarring permits the rapid invasion of immune and neural cells to repopulate the tissue void: (3) a remodeling of the bioscaffold and newly deposited ECM is required (4) to allow the establishment of a tissue architecture that matures and restores function (Figure 6). However, these phases merely provide a general descriptive framework that needs to be refined by detailed mechanistic studies to elucidate how each cell type influences the overall process. Considering the range and dynamics of molecules each cell produces, this poses a formidable challenge.

## Conclusion

With the discovery of adult neurogenesis, Ramon y Cajal’s decree on the lack of regeneration in the CNS has been undergoing a rigorous reassessment. Although spontaneous tissue regeneration does not occur in the mammalian CNS (Illis, 2012), engineering strategies are gradually overcoming the biological and physical challenges imposed by brain injury through harnessing the potential of endogenous neurogenesis (Modo and Badylak, 2019). In this context, it is important to contrast conditions with volumetric tissue loss (i.e., a chunk of tissue is missing) versus neurological disease in which tissue gradually shrinks (i.e., tissue atrophy). We here described putative mechanisms involved in tissue regeneration after a volumetric loss of brain tissue. Some of these strategies, such as enhancing endogenous neurogenesis, can potentially provide benefits to both scenarios, but provision of, for instance, a scaffold, will only be amenable to conditions in which a tissue cavity formed. In a clinical scenario, it can be expected that a patient will receive rehabilitation training shortly after a stroke and this could be combined with an enhancement of neurogenesis. Bioscaffold implantation would only occur after cavitation is complete, with concomitant neurogenesis and rehabilitation training being required to yield an optimal outcome. Although the aim is to define specific modes and mechanisms of action, we have to recognize that these at present can merely be putative. Investigations first have to define zero and first orders of approximation of the variables and conditions required to produce brain tissue regeneration. Consequently, the therapeutic value of restoring lost tissue currently remains unclear. Although fetal tissue transplant suggest that *de novo* brain tissue can integrate into the host and reduce behavioral impairments, we currently have a poor understanding of what cellular substrates are required to support behavioral changes. Studies in axolotl suggest that regenerated tissue is unlikely to exactly replicate the tissue structure that emerged through brain development, but might support restoration of function. Still, a functional tissue requires integration with veterate brain regions to support behavior and cognition. The utility of brain tissue regeneration is not to perfectly restore the damaged brain, but to produce a sufficient tissue substrate that can reduce behavioral deficits. It is expected that this review provides the context for future studies to improve engineering strategies that will produce a robust regeneration of brain tissue.

## Author Contributions

The author confirms being the sole contributor of this work and has approved it for publication.

## Conflict of Interest

The author declares that the research was conducted in the absence of any commercial or financial relationships that could be construed as a potential conflict of interest.
